# Pharmacometabolomics of Meglumine Antimoniate in Patients With Cutaneous Leishmaniasis

**DOI:** 10.3389/fphar.2019.00657

**Published:** 2019-06-20

**Authors:** Deninson Alejandro Vargas, Miguel Dario Prieto, Alvaro José Martínez-Valencia, Alexandra Cossio, Karl E. V. Burgess, Richard J.S. Burchmore, María Adelaida Gómez

**Affiliations:** ^1^Centro Internacional de Entrenamiento e Investigaciones Médicas, CIDEIM, Cali, Colombia; ^2^Universidad de Valle, Cali, Colombia; ^3^Universidad ICESI, Cali, Colombia; ^4^Glasgow Polyomics, Wolfson Wohl Cancer Research Centre, College of Medical Veterinary & Life Sciences, University of Glasgow, Glasgow, United Kingdom; ^5^Institute of Infection, Immunity and Inflammation, College of Medical, Veterinary and Life Sciences, University of Glasgow, Glasgow, United Kingdom

**Keywords:** cutaneous leishmaniasis, meglumine antimoniate, pharmacometabolomics, biomarkers, allantoin, taurine

## Abstract

Control of cutaneous leishmaniasis (CL) in the Americas is dependent on chemotherapy with parenteral pentavalent antimonials. High rates of treatment failure urge the search for predictive and prognostic markers of therapeutic responsiveness. In this study, we aimed to identify biomarkers of therapeutic response during treatment with meglumine antimoniate (MA). We conducted untargeted metabolomic profiling of plasma samples from CL patients (n = 39; 25 who cured and 14 who did not cure), obtained before and at the end of treatment. Exposure to MA induced metabolic perturbations primarily reflecting alteration in long-chain fatty acid β-oxidation and energy production. Allantoin, *N*-acetylglutamine, taurine, and pyruvate were significantly more abundant in samples from patients who responded to treatment, and were predictive and prognostic of treatment outcome in this patient cohort (AUC > 0.7). In an *ex vivo* model of infection, allantoin but not taurine enhanced the MA-dependent killing of intracellular *Leishmania* (*Viannia*) *panamensis*. Our results support the participation of metabolites mediating antioxidant and wound healing responses in clinical cure of CL, revealing relationships between metabolism and immune responses in the outcome of antileishmanial treatment.

## Introduction

Cutaneous leishmaniasis (CL) is endemic in more than 90 countries and 1 million new cases are estimated to be globally reported each year. Control of CL in the Americas is contingent upon active case detection and treatment. Although pentavalent antimonials (Sb^V^) remain the first-line treatment option, therapeutic failure rates of 19% to 75% challenge its usefulness (Palacios et al., 2001; Vélez et al., 2010). Adverse drug reactions (ADRs) have been reported to occur in up to 64% of individuals treated with these drugs (Oliveira et al., 2011), and can result in acute pancreatitis, hepatotoxicity, and death in most severe cases (Oliveira et al., 2011). The high rates of treatment failure and of ADR urge the identification of readily accessible and objectively measured biomarkers of therapeutic response and toxicity.

Populations most affected by CL in the Americas are inhabitants of rural dispersed communities with limited access to health care systems. The extended time for patient follow-up required to determine the therapeutic response (3 to 6 months) (Olliaro et al., 2013) constrains clinical follow-up and pharmacovigilance strategies. Identification of prognostic biomarkers for early definition of the therapeutic response could contribute to mitigate these limitations.

Pharmacometabolomic approaches have contributed to understanding the mechanism of action of drugs through identification of metabolic signatures associated with therapeutic responses (Johnson et al., 2012). Although scantly applied in neglected tropical diseases research, metabolomics has been recently used to explore the mechanisms of antimonial drug resistance in *in vitro* cultures of *Leishmania donovani* and *Leishmania infantum*; polyamides and trypanothione biosynthetic pathways were identified as contributors to the resistance phenotype (Canuto et al., 2012; Rojo et al., 2015), concurring with previous findings at the genome and proteome levels (Ouellette et al., 2004).

The feasibility of obtaining human samples for metabolomics such as serum, plasma, saliva, or urine, and the lower complexity of data matrices resulting from the smaller dimension of the metabolome compared with the genome (from ∼30,000 genes to ∼2,500 metabolites) strongly support its application in the pipeline of biomarker discovery (Johnson et al., 2016). The use of metabolomics toward therapeutics for neglected infectious diseases, such as leishmaniasis, has been underexploited. Only recently, Vincent et al. (2016) have documented the implementation of a liquid chromatography mass spectrometry (LC-MS) metabolic approach to identified biomarkers for diagnosis and definition of disease stage in human African trypanosomiasis. In this study we implemented an unbiased exploratory metabolomics approach to identify predictive and prognostic candidate biomarkers of the outcome of treatment with meglumine antimoniate (MA) in samples from patients with CL caused by *L. Viannia*, as well as to describe the metabolomic perturbations associated to drug exposure.

## Materials and Methods

### Ethics Statement

This study was approved and monitored by the Institutional Review Board for Ethical Conduct of Research Involving Human Subjects of CIDEIM (approval code CIEIH 1209), in accordance with national and international guidelines for conduct of clinical studies. All individuals voluntarily participated in the study; each participant signed the informed written consent at recruitment.

### Study Design and Participants

**Figure 1:**
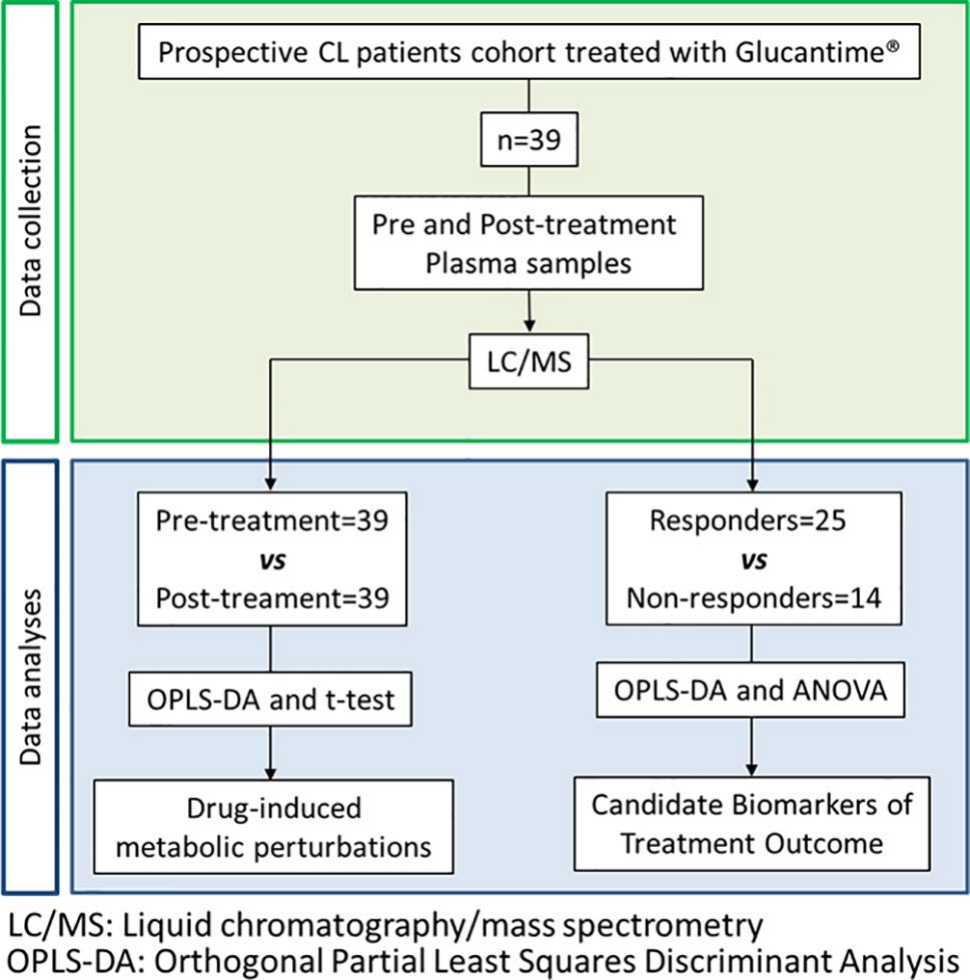
Flowchart of the study design.

This metabolomics study was designed as a biomarker discovery study, aimed to determine the metabolic perturbations associated with exposure to antimonial drugs in CL patients, and to identify candidate biomarkers of antimonial treatment outcome in plasma samples (**Figure 1**). Parasitologically diagnosed CL patients (n = 39), 18 to 47 years of age, with time of lesion evolution <6 months and who had a reported overall adherence to treatment with MA > 85% were included between years 2012 to 2015 (**Table 1**). Peripheral blood samples were taken before initiation of treatment and at the end of treatment from all participants. Healthy and non-treated controls were not included as the purpose of this study was to identify biomarkers of therapeutic responsiveness in infected patients and not biomarkers of infection. Sample size could not be calculated as no prior data was available for probabilistic estimations. Therefore, sample size for our discovery study was defined based on the well-established biomarker pipeline and respective sample dimensions: discovery studies (unbiased semiquantitative studies of small sample size—within the tens of samples), qualification studies (target-driven quantitative small sample size studies—tens of samples), verification studies (targeted quantitative studies with larger cohorts, usually within the hundreds of samples), and validation studies (cohort studies within the thousands of samples) (Rifai et al., 2006).

**Table 1:** Clinical and demographic characteristics of study participants.

Characteristic	Total	Responders	Nonresponders	p value*
Number of participants	39 (100)	25 (64)	14 (36)	–
Sex, n (%)				
Male	36 (92)	25 (100)	11 (78.6)	0.039^‡^
Female	3 (8)	–	3 (21.4)	
Age in years, median (range)	30 (18–47)	30 (18–45)	29.5 (19–47)	0.735^§^
Ethnicity, n (%)				
African descent	37 (95)	25 (100)	12 (85.7)	0.122^‡^
Other	2 (5)	–	2 (1.3)	
Weight, mean (SD), kg	69.4 (8.4)	67.6 (7.4)	72.4 (9.6)	0.092^§^
Time of disease evolution (months), median (range)	1 (1–5)	2 (1–5)	1 (1–5)	0.311^§^
Number of lesions per patient, median (range)	2 (1–6)	1 (1–5)	2 (1–6)	0.634^§^
Percentage of treatment adherence, median (range)^†^	100 (85–100)	100 (98–100)	100 (85–100)	0.075^‡^
Geographical area of reported infection (Department). n (%)				
Nariño	32 (82.1)	20 (80)	12 (85.7)	0.655^‡^
Other	7 (17.9)	5 (20)	2 (14.3)	
Isolated *Leishmania* species, n(%)				
*L. V. panamensis*	31 (79.5)	19 (76)	12 (85.7)	0.813^‡^
*L. V. braziliensis*	2 (5.1)	2 (8)	0	
Not available or contaminated	6 (15.4)	4 (16)	2 (14.3)	
**Adverse Drug Reactions (ADR)**				
Patients reporting adverse drug reactions, n (%)				
None	12 (30.8)	6 (24)	6 (42.9)	0.286^‡^
At least one	27 (69.2)	19 (76)	8 (57.1)	
Intensity of ADR, n (%)				
Mild	24 (89)	17 (89)	7 (87)	1.000^‡^
Moderate	3 (11)	2 (11)	1 (13)	
Type of reaction, n (%)				
Fever	18 (26.1)	12 (29.3)	6 (21.4)	0.480^‡^
Pain at the injection site	9 (13.0)	3 (7.3)	6 (21.4)	
Headache	9 (13.0)	7 (17.1)	2 (7.1)	
Arthralgia	9 (13.0)	5 (12.1)	4 (14.3)	
Fatigue	5 (7.2)	3 (7.2)	2 (7.1)	
Myalgia	5 (7.2)	4 (9.8)	1 (3.6)	
Others	14 (20.3)	7 (17.1)	7 (25.0)	

*Contrast between responders and non-responders. ^†^Based on ampules received vs. ampules ordered. ^‡^Chi2/Fisher. ^§^T-test/Wilcoxon rank sum test.

Patients received standard-of-care treatment with MA (20 mg/kg/day for 20 days) and clinical evaluations were conducted at the end of treatment and 13 weeks after initiation of treatment, time at which clinical outcome was determined. Peripheral blood samples for isolation of plasma were obtained before initiation of treatment and within 8 days after end of treatment. The clinical outcome was defined according to the most recent harmonization guidelines for CL (Olliaro et al., 2013, Olliaro et al., 2018). Cure was defined as complete reepithelialization and absence of inflammatory signs for all lesions. Treatment failure was defined as incomplete reepithelialization and/or presence of induration, raised borders, or other evidence of inflammation of any CL lesion at the end of follow-up (90 days after initiation of treatment); or reactivation of the original lesion(s); or appearance of new lesions during follow-up. Safety monitoring was performed and recorded during the follow-up visits. Adverse events (AE), were recorded and standardized (by name and severity) according to the Common Toxicity Criteria of the National Cancer Institute, V 4.0 (National Cancer Institute, 2009). Causality was established using WHO-UMC criteria and the Naranjo algorithm (Naranjo et al., 1981) and were reported as possibly, probably, or definitely related to treatment with MA (**Table 1**), and thus considered ADRs. Quality control of clinical data included double data entry from paper case report forms (CRF) to a Microsoft Office Access database, external data monitoring, and resolution of discrepancies by consensus.

### Sample Processing and Metabolite Extraction

Plasma was obtained from heparin anticoagulated peripheral blood samples by centrifugation at 800 × *g* for 10 min. For metabolite extraction we adapted the protocol described by Vincent et al. (2012), and all samples were processed in a single batch by the same person in the same day. Briefly, 100 μl of plasma was mixed with chloroform/methanol (in a 1:3 ratio), vortexed on a cooled (4°C) mixer for 1 h, centrifuged for 10 min at 14,000 × *g* at 4°C, and the supernatant transferred and stored at −80°C.

### LC/MS Data Acquisition and Processing

Samples were analyzed by hydrophilic interaction LC/MS (UltiMate 3000 RSLC, Thermo Fisher) using a 150 × 4.6 mm ZIC-pHILIC column (Merck SeQuant) running at 300 µl/min and Orbitrap Exactive (Thermo Fisher) detection. Mass spectrometer parameters were 50,000 resolving power in positive/negative switching mode. Electrospray ionization (ESI) voltage was 4.5 kV in positive and 3 kV in negative modes. Buffers consisted of (A) 20 mM ammonium carbonate (Sigma) in H_2_O and (B) Merck SeQuant: acetonitrile (Rathburn Chemicals). The gradient ran from 20% A: 80% B to 80% A: 20% B in 15 min, followed by a wash at 95% A: 5% B for 3 min, and equilibration at 20% A: 80% B for 5 min.

Raw mass spectrometry data were processed using XCMS (Smith et al., 2006), MzMatch (Scheltema et al., 2011), and in-house R-scripts for deep filtering, post-processing, and identification. Peaks were visualized using PeakML Viewer (Scheltema et al., 2011). The data were compiled using the IDEOM software (Creek et al., 2012). Classification of metabolite annotations followed the MSI guidelines (Sumner et al., 2007). Compounds annotated on the basis of a <3 ppm accurate mass threshold, searched against the IDEOM database, were described as “annotations,“ while compounds matched to an authentic standard with <3 ppm accurate mass error and <5% retention time deviation were classed as “identifications.”

### Quality Control of LC/MS Data

Aliquots from each sample were pooled to generate a quality control (QC) master sample. All samples were analyzed in 1 day and in one batch in a randomized manner. Stock solutions of 116 authentic standard compounds were prepared in ethanol, 50% ethanol/water, or Milli-Q water, depending on solubility (**Figure S1**). A working solution containing all authentic standards was prepared and run at the beginning and at the end of the run (Creek et al., 2011). The QC was injected before starting the LC/MS run and every five samples for stable run assurance. The quality of the chromatography and signal reproducibility was checked by analysis of QC samples, internal authentic standards, and total ion chromatograms. Samples that displayed unacceptable analytical variation (retention time drift) were removed from further analysis. Metabolites with more than 20% missing values, confidence identification <5, and drug derivatives were excluded from the analysis. Additional manual curation was performed on all datasets confirming identification and removing false identifications based on peak quality.

### Data Curation and Statistical Analysis

Clinical and demographic data were analyzed using R software version 3.3.2 and STATA 14, using untransformed data. For metabolomic data, principal component analysis (PCA) analysis was used to identify outlier samples. MetaboAnalyst 4.0 (Chong et al., 2018), XCMS, and GraphPad Prism 6 were used for data analysis and interpretation. Peak intensity table was normalized by probabilistic quotient normalization (Dieterle et al., 2006), generalized log transformed, and Pareto scaled (van den Berg et al., 2006). A statistical pipeline based on univariate (Student’s t-test and ANOVA) and multivariate analyses [orthogonal partial least squares discriminant analyses (OPLS-DA)] was used to identify metabolites associated with drug exposure, toxicity, and treatment outcome. OPLS-DA statistics R^2^Y and Q^2^ represent the total sum of variation in Y explained by the model, and the goodness of prediction calculated by full cross-validation, respectively (Worley and Powers, 2012). KEGG, MetaboAnalyst 4.0, and text mining were used for pathway analyses (Chong et al., 2018). ROC curves were constructed by logistic regression.

### Cytotoxicity Assay

Cytotoxicity of candidate metabolites was evaluated in THP-1 cells in dose response experiments: allantoin (0.005 mM–31 mM) and turine (0.05 mM–10 mM). The range of allantoin doses was selected based on its maximum solubility in RPMI medium (≈31 mM) and FDA-approved doses for local therapy as a skin protectant (≈31–126 mM) (FDA, 1978). Taurine concentrations were based on those found in plasma in healthy individuals (Marcinkiewicz and Kontny, 2014) and those reported as cytoprotective concentrations (Eppler and Dawson, 2002). One hundred thousand cells were seeded in a 96-well plate in RPMI medium supplemented with 10% FBS and incubated at 37°C, 5% CO_2_ with allantoin or taurine for 72 h. Cytotoxicity was evaluated using the MTT (3-(4,5-dimethylthiazol-2-yl)-​2,5-diphenyltetrazolium bromide) method following the manufacturer’s instructions (ATCC) (**Figure S2**).

### PBMCs Isolation, *Ex Vivo* Infection, and Parasite Survival Assays

Blood samples were collected from six CL patients before initiation of treatment. Peripheral blood mononuclear cells (PBMCs) were isolated by centrifugation over a Histopaque 1077 gradient (Sigma-Aldrich). PBMCs were resuspended in RPMI with 10% FBS and seeded in 96-well plates. Cells were infected in a 1:10 parasite-to-monocyte ratio for 24 h with serum-opsonized *L.V. panamensis* (transfected with the luciferase reporter gene) as previously optimized and described (Gonzalez-Fajardo et al., 2015). Taurine or allantoin (Sigma-Aldrich) were added alone or in combination (simultaneous exposure) with 4 μg/ml MA for 72 h at 34°C, 5% CO_2_, and parasite burden was evaluated by luminometry (Gonzalez-Fajardo et al., 2015).

## Results

### Clinical and Demographic Characteristics of Study Participants

A total of 39 participants were included in this study, 25 who responded to MA treatment and 14 who did not respond (**Table 1**, **Figure 1**). The majority of participants (92%) were young adult males of Afro-Colombian descent from the department of Nariño, Colombia. No significant differences were found in demographic or clinical characteristics between groups, with the exception of gender where the only three female participants were recruited to the treatment failure group (p = 0.04). At least one ADR was found in 27 participants (69%), the most common ones being fever (18.4%) followed by pain at the injection site (9.2%) and headache (9.2%) (**Table 1**). No serious adverse events were reported.

### Exposure to MA Alters the Plasma Metabolome of CL Patients

Based on the raw metabolite data, metabolomes from four patients (three responders and one of the female non-responders) were excluded from further analyses; two of them had poor quality peak spectra, while the other two were identified as outliers in the PCA (**Figure S3**). No statistical differences in clinical and demographic variables were found among groups after outlier exclusion. After data curation and quality control, from a total of 11,309 features, 536 metabolites were detected in plasma samples and 48 metabolites were identified against authentic pure standards (**Dataset S1**). The remaining 488 metabolites were classified as annotated. *N*-methyl glucamine (meglumine), a by-product of MA, was detected in all post-treatment samples (relative mean intensity 26.734) serving as an indicator of drug exposure in the study population; this metabolite was excluded from further analysis.

**Figure 2:**
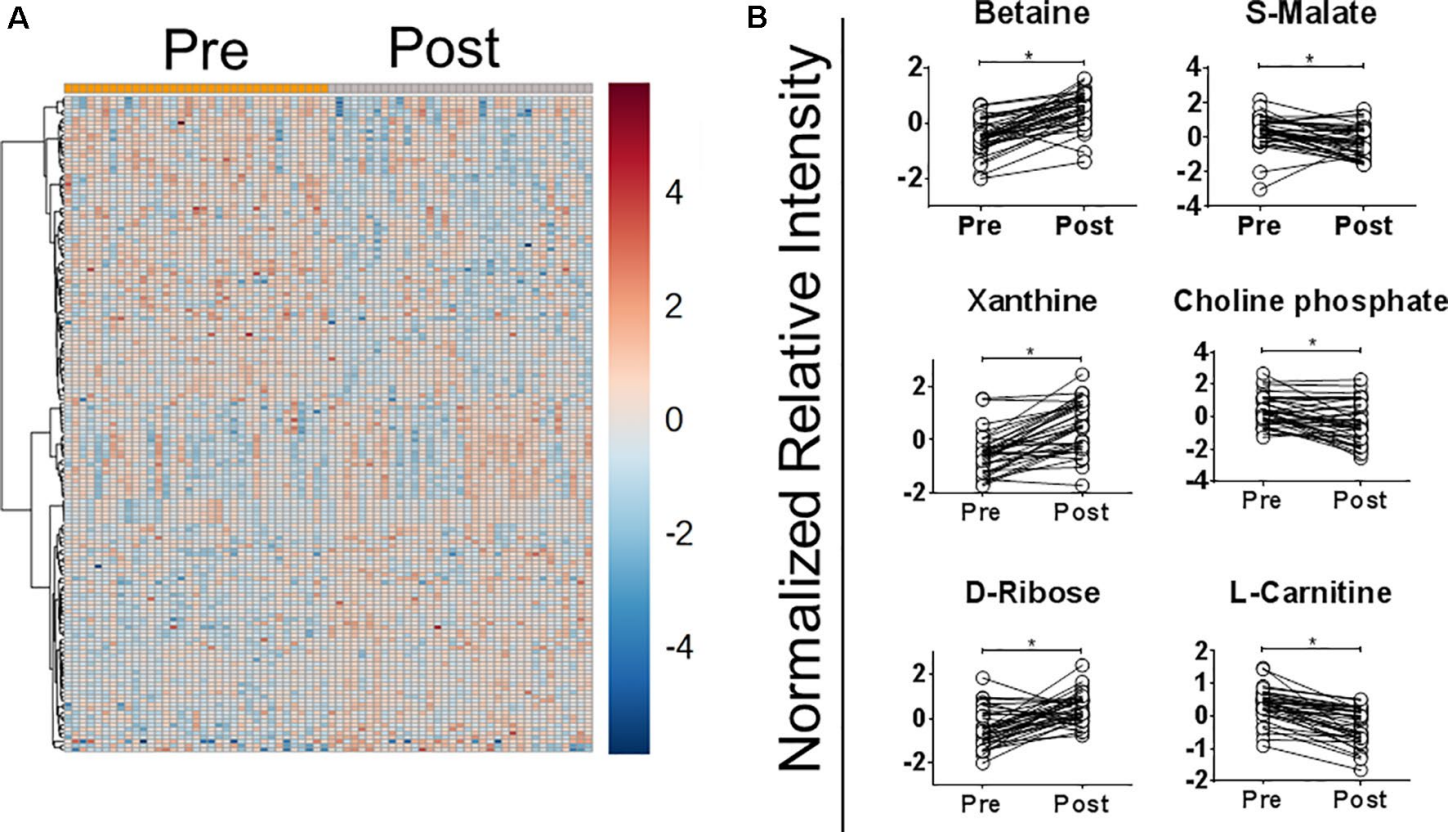
Exposure to meglumine antimoniate alters the plasma metabolome of CL patients. Plasma samples from CL patients (n = 35) obtained before (Pre) and at the end of treatment (Post) with MA were analyzed by LC/MS. **(A)** Heatmap representation of the normalized relative intensity of metabolites with significantly different abundance between samples obtained before and at the end of treatment, built using Euclidean distance and ward clustering algorithm. **(B)** Paired dot plot of identified metabolites significantly modulated after treatment with MA. Data were normalized using the pooled sample from the pre-treatment group. Statistical significance in dot plots was determined using paired t-test. *p <0.0.

The abundance of 30.4% of detected metabolites (163 of 536) was significantly modified after *in vivo* exposure to MA [paired t-test p ≤ 0.05, false discovery rate (FDR) ≤0.1], 19 of which were identified against an authentic pure standard. Fifty-three percent (88 of 163) were more abundant after treatment (**Figure 2A** and **Dataset S2**). To select metabolites that were consistently modified after antimonial drug exposure, data were analyzed by OPLS-DA. Of the 163 differentially abundant metabolites found by paired t-test analysis, 38 were also discriminatory by OPLS-DA (sum R^2^Y = 0.93 and cross-validation test Q^2^ = 0.62; cutoff thresholds of 1.5 ≤ p[1] ≤ -1.5 and 0.3 ≤ p (corr)[1] ≤ -0.3 in the S-plot; **Dataset S2** and **Figure S4**). Among these, six metabolites were identified: betaine, choline phosphate, D-ribose, L-carnitine, S-malate, and xanthine (**Figure 2B**). The relative abundance of betaine, xanthine, and D-ribose increased after treatment, while S-malate, choline phosphate, and L-carnitine decreased. Both annotated and identified metabolites were included for metabolic pathway analysis, showing that metabolites within the pathways of long-chain fatty acid metabolism and β-oxidation (L-carnitine and choline phosphate, 3-methylglutarylcarnitine, and cis-5-tetradecenoylcarnitine), redox balance (choline phosphate, guanidinoacetate, S-malate, L-dehydroascorbate, and betaine), and nucleotide metabolism (S-dihydroorotate, 3-methylguanine, D-ribose and xanthine) were significantly and differentially abundant after MA exposure (**Dataset S2**).

### Metabolites Associated With Wound Healing and Redox Balance Are Differentially Abundant in Responders and Non-Responders

**Figure 3:**
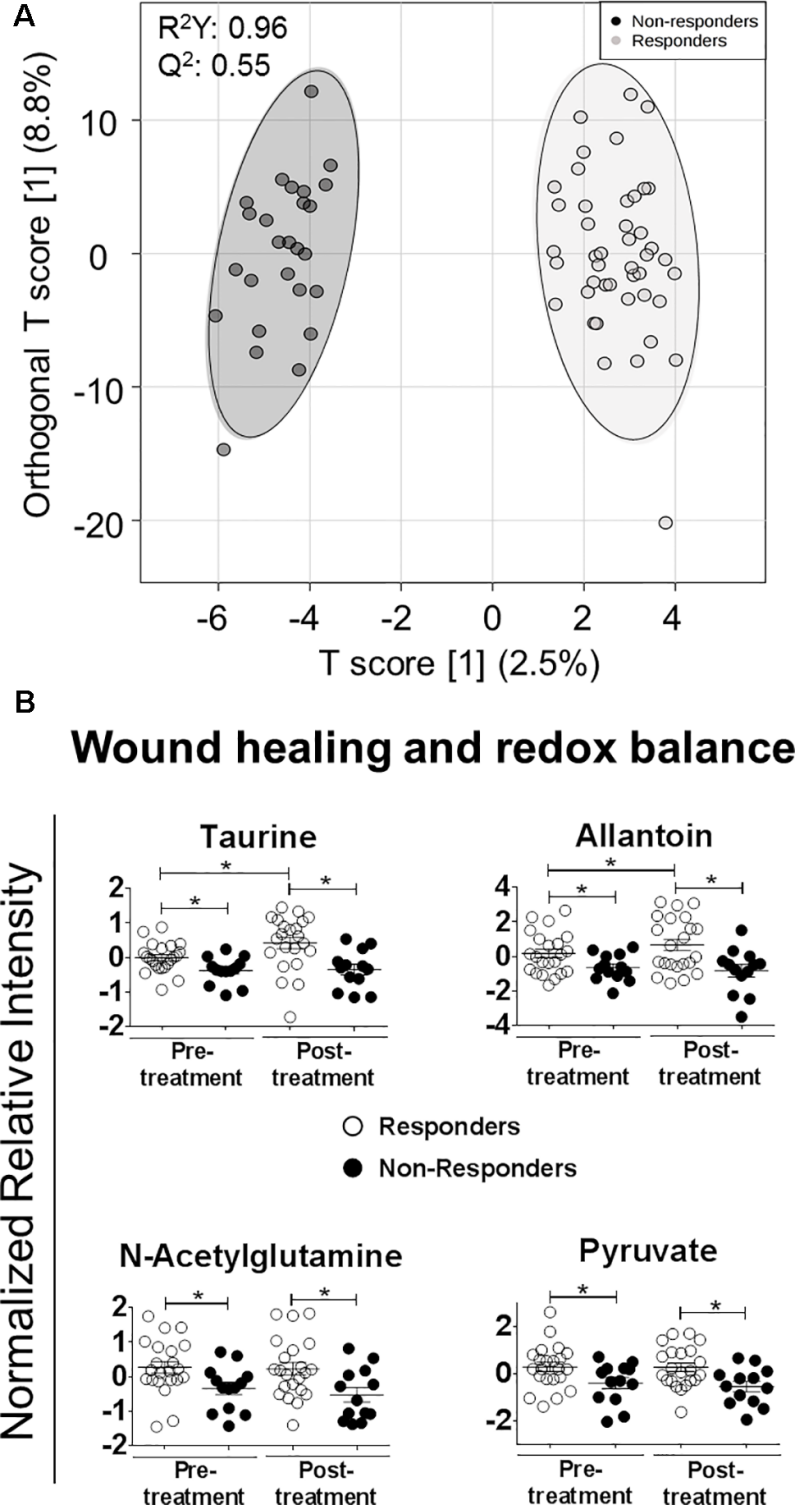
Metabolites associated with wound healing and redox balance are more abundant in CL patients who cured. **(A)** OPLS-DA model of metabolites depicts the separation of all samples (pre- and post-treatment) in each group (responders and non-responders), each circle representing an individual sample. **(B)** Scatter dot plots of selected metabolites differentially abundant between responders and non-responders. Whiskers represent the 5–95 percentile. Data were normalized using the pooled sample from plasmas of the responders group. Statistical significance was determined using t-test. *p <0.05.

An OPLS-DA model was constructed to select metabolites that could discriminate between patients that cured (responders, n = 22) and patients that did not cure (non-responders, n = 13). Pre-treatment and end-of-treatment plasma samples were included for this analysis. The model allowed separation of samples from responders and non-responders with a sum R^2^Y: 0.96 and cross-validation test Q^2^: 0.55 (**Figure 3A**). Cutoff thresholds of 0.65 ≤ p[1] ≤ -0.65 and 0.3 ≤ p (corr)[1] ≤ -0.3 in the S-plot were defined for selection of the most discriminatory metabolites. Of 30 metabolites with discriminatory potential, 26 were annotated and 4 were identified against an authentic pure standard (**Dataset S3**).

A four-way ANOVA with Fisher´s LSD *post hoc* analysis was conducted to strengthen selection of candidate metabolites predictive and prognostic of the therapeutic response (**Dataset S3**). In this analysis, 30 metabolites could discriminate responders from non-responders, 4 of these exclusively in pre-treatment samples (p ≤ 0.05, FDR ≤ 0.1), 11 exclusively in post-treatment samples, and 15 in both pre- and post-treatment plasma samples (**Dataset S3**). Seventeen differentially abundant metabolites were also found as discriminatory by OPLS-DA. Of these, 13 were annotated and 4 identified (**Table 2**).

**Table 2:** Plasma metabolites with significant differences in CL patients that responded or did not respond to MA treatment.

Metabolites^‡^	Elemental formula	Mass (Da)	RT (min)	*p* value	FDR	*Post hoc* Fisher’s LSD*
**Taurine^§^**	C_2_H_7_NO_3_S	125.01	13.55	0.00028	0.00948	1-2; 3-4
**Allantoin^§^**	C_4_H_6_N_4_O_3_	158.04	13.28	0.00324	0.05261	1-2; 3-4
***N*-acetylglutamine^§^**	C_7_H_12_N_2_O_4_	188.08	10.57	0.00805	0.09197	1-2; 3-4
**Pyruvate^§^**	C_3_H_4_O_3_	88.016	7.93	0.01005	0.09606	1-2; 3-4
D-erythrose	C_4_H_8_O_4_	120.04	10.34	0.00012	0.00600	1-2; 3-4
Taurochenodeoxycholate-3-sulfate	C_26_H_45_NO_9_S_2_	289.63	4.34	0.00017	0.00723	1-2; 3-4
Ala-Lys-Ser-Arg	C_18_H_36_N_8_O_6_	230.14	13.27	0.00019	0.00743	1-2; 3-4
Glycochenodeoxycholate 7-sulfate	C_26_H_43_NO_8_S	264.64	4.66	0.00038	0.01079	3-4
2-Hydroxypyridine	C_5_H_5_NO	95.04	7.98	0.00076	0.01951	1-2; 3-4
FA methyl jasmonate	C_13_H_20_O_3_	224.14	3.94	0.00162	0.03192	1-2; 3-4
N6-methyl-L-lysine	C_7_H_16_N_2_O_2_	160.12	19.26	0.00427	0.06468	1-2; 3-4
ethylpyruvate	C_5_H_8_O3	116.05	5.34	0.00435	0.06468	1-2; 3-4
Fatty acyls^¶^	C_13_H_18_O_5_	254.12	5.25	0.00459	0.06650	1-2; 3-4
Glu-Phe-Trp	C_25_H_28_N_4_O_6_	240.1	7.71	0.00558	0.07844	1-2; 3-4
Sterol lipids^||^	C_24_H_40_O4	392.29	4.83	0.00855	0.09197	1-2
N5-ethyl-L-glutamine	C_7_H_14_N_2_O_3_	174.1	12.66	0.00900	0.09197	1-2; 3-4
Stearoylcarnitine	C_25_H_49_NO_4_	427.37	4.72	0.00945	0.09197	3-4

^‡^Data was normalized using a pooled sample from responders, gLog_2_ transformed and pareto scaled. RT: retention time in minutes, mass in Daltons.

^*^Group contrasts with statistical significance in the Fisher LSD post hoc: 1: responders pre-treatment, 2: non-responders pre-treatment, 3: responders post-treatment, 4: non-responders post-treatment.

^§^ **In bold**, metabolites identified against an authentic pure standard.

^¶^[FA methyl_hydroxyl_oxo(5:2/4:0)] methyl 4-[2-(2-formyl-vinyl)-3-hydroxy-5-oxo-cyclopentyl]-butanoate.

^||^[ST hydrox] 3alpha,7alpha-dihydroxy-5beta-cholan-24-oic acid.

**Figure 4:**
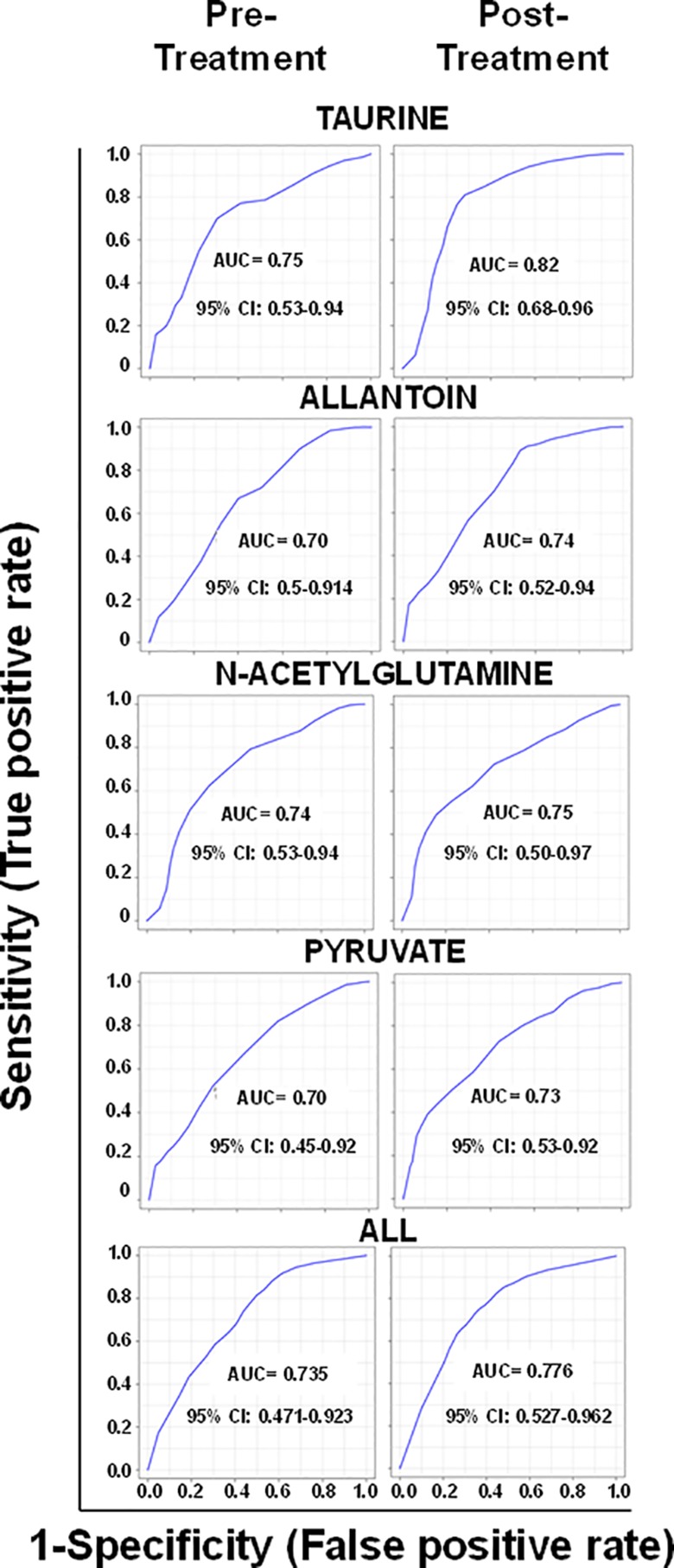
Metabolites with predictive and prognostic potential of treatment outcome. ROC curves represent the area under the curve (AUC) of the false-positive rate vs. the true positive rate. Data from all samples for each individual metabolite, or the combination of the four selected metabolites (ALL), were used to construct the ROC curves. ROC curves of metabolites identified in pre-treatment samples are shown in the left and in post-treatment samples in the right. CI: confidence interval.

Of the annotated metabolites, six were related to lipid metabolism (two fatty acyls, taurochenodeoxy-cholate-3-sulfate, glycochenodeoxycholate 7-sulfate, sterol lipids, and stearoylcarnitine), two with energy metabolism (D-erythrose and ethylpyruvate), and five with amino acid metabolism (N5-ethyl-L-glutamine, N6-methyl-L-lysine, 2 hydroxypyridine, Glu-Phe-Trp, and Ala-Lys-Ser-Arg) (**Table 2**). All identified metabolites (taurine, *N*-acetylglutamine, allantoin, and pyruvate) participate in antioxidant and wound healing responses, and were consistently found more abundant in responders both in pre- and post-treatment samples (**Figure 3B**). Among identified metabolites, the abundance of taurine and allantoin significantly increased at the end of treatment in patients who responded to treatment, in contrast to non-responders (**Figure 3B**). Allantoin, *N*-acetylglutamine, taurine, and pyruvate had both predictive and prognostic potential as determined by ROC curves of data from pre-treatment and post-treatment samples, respectively (AUC >0.7) (**Figure 4**). ROC curve analyses of the individual metabolites or a composite metabolic signature including allantoin, *N*-acetylglutamine, taurine, and pyruvate showed that taurine had the highest predictive and prognostic potential in our patient cohort (AUC = 0.75 and AUC = 0.82, respectively) (**Figure 4**).

### Allantoin Promotes MA-Dependent Killing of *L.V. Panamensis*

**Figure 5:**
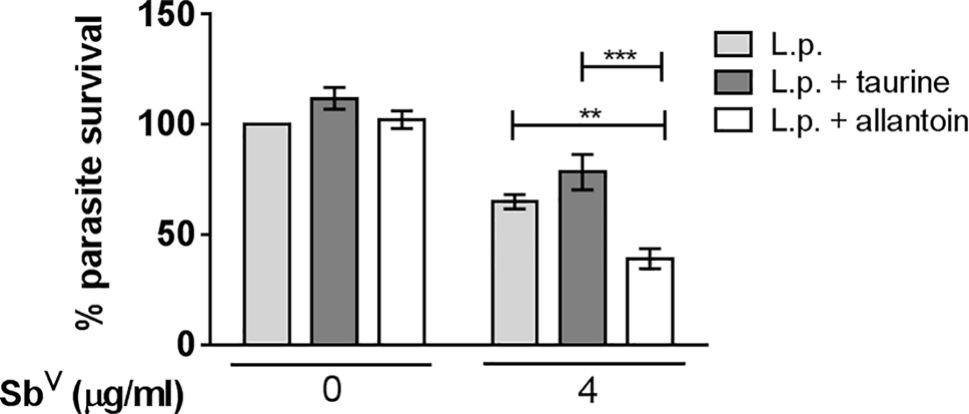
Allantoin potentiates antimony-mediated killing of intracellular *Leishmania*. Peripheral blood mononuclear cells from six CL patients were infected *ex vivo* with *L. V. panamensis* (L.p.) for 24 h. Taurine (10 mM) or allantoin (10 mM) was added alone or in combination with MA (4 μg/ml) for 72 h, and parasite burden was evaluated by luminometry. Shown is the percent parasite survival relative to infected and untreated cells. Statistical significance was determined by t-test. *p < 0.05.

Allantoin and taurine have been described as important mediators of wound healing and participate in antioxidant responses (Araújo et al., 2010; Eppler and Dawson, 2002; Macalister, 1912). Based on this, we explored whether these metabolites could enhance the antimony-mediated killing of *Leishmania*. PBMCs from CL patients were infected *ex vivo* with *L. V. panamensis* and exposed to 10 mM taurine or 10 mM allantoin alone or in combination with MA. Selection of these doses was based on concentrations not cytotoxic for cells, and in the case of allantoin we also consider the minimum dose allowed by FDA for use as a topical treatment and its solubility in RPMI medium. Exposure to taurine or allantion alone, or taurine in combination with MA, did not modify the parasite burden of infected PBMCs. However, when infected cells were exposed to allantoin in combination with MA (4 µg/mL), parasite killing was significantly enhanced compared to the MA-only control (**Figure 5**), indicating a potential contribution of allantoin in antimony-mediated parasite killing.

## Discussion

Despite the high rates of treatment failure and ADRs, systemic pentavalent antimonials remain the first-line treatment for CL. Unnecessary exposure of patients to these highly toxic drugs should be minimized, and thus identification of predictive biomarkers of treatment outcome is a priority in the clinical management of CL. Assessment of therapeutic response for CL is conducted at 3 and 6 months after initiation of treatment. However, the occurrence of CL in dispersed rural settings constrains access to treatment, treatment follow-up, and pharmacovigilance actions. Thus, early predictors of the therapeutic response (predictive and prognostic biomarkers) could support public health interventions and clinical practice. In this study, we provide the first metabolomic profiles of CL patients undergoing treatment with pentavalent antimonials, and report a set of predictive and prognostic candidate biomarkers of therapeutic outcome to be scaled toward biomarker validation studies. Increased levels of allantoin, taurine, and *N*-acetylglutamine, metabolites involved in antioxidant and wound healing responses, were detected before and at the end of treatment in patients who cured. This supports the fact that induction of host immune/wound healing responses during treatment, in addition to controlling the parasite burden, is central to therapeutic cure, and furthermore highlights a previously unrecognized participation of antioxidant mechanisms in healing of CL.

Exposure to metalloids such as antimony results in disturbance of ATP production, induction of oxidative and osmotic stress (in part due to production of reactive oxygen species), lipid peroxidation, and downstream loss of membrane integrity (Gebel, 1997; Pulido, 2003; Wyllie and Fairlamb, 2006; Bento et al., 2013). Drug-induced perturbation of metabolites associated with redox balance is reflected by the significant increase in betaine, D-ribose, and xanthine and decrease of choline phosphate and S-malate at the end of treatment. Betaine protects against osmotic stress (Obeid, 2013) and redox imbalances primarily through homocysteine conversion to methionine (Ganesan et al., 2011). Reduced abundance of choline phosphate is consistent with its oxidation to betaine and with the higher levels of betaine found after treatment with MA. D-ribose and xanthine are metabolites within purine biosynthetic pathways. Their higher abundance after drug treatment could contribute to protection against metal-induced oxidative stress through the oxidative branch of the pentose phosphate pathway by production of NADPH and purine degradation to urate, the major antioxidant in the blood (Patra and Hay, 2014). These results provide evidence that the plasma metabolome of CL patients exposed to MA reflects a response against the oxidative stress induced by antimonial drugs.

The mechanisms of action of antimonials are not fully understood (Croft et al., 2006; Gebel, 1997; Oliveira et al., 2011). It has been shown that exposure to antimonials leads to inhibition of fatty acid β-oxidation, and this has been considered part of the mechanism(s) of antileishmanial action (Berman et al., 1987; Croft et al., 2006). However, how these drugs modulate fatty acid β-oxidation is still unknown. Our results showed that L-carnitine, a facilitator of fatty acid transport to the mitochondria for β-oxidation (Vaz and Wanders, 2002), was less abundant at the end of treatment in CL patients, compared to pre-treatment samples. Concordantly, other acylcarnitine intermediates (3-methylglutarylcarnitine and cis-5-tetradecenoylcarnitine) associated with dysfunction of normal fatty acid β-oxidation (Roe et al., 1986; Wood et al., 2001; Vaz and Wanders, 2002; Rinaldo et al., 2008) were increased after MA treatment. These results suggest that reduced lipid transport to the mitochondria could contribute to the MA-induced perturbations in fatty acid β-oxidation or metabolic adaptations to the stressor. Although these inferences derive from observations of human plasma samples and reflect the functions of host cells, these same mechanisms could also be operating in *Leishmania* cells; however, this remains to be demonstrated.

In our study, plasma metabolites involved in inflammatory and oxidative stress pathways were identified as candidate biomarkers of the outcome of treatment. ROC curve analyses showed that taurine, allantoin, pyruvate, and *N*-acetylglutamine had the potential to be both predictive and prognostic biomarkers in our patient cohort. *N*-acetylglutamine, allantoin, and taurine have important roles in the control of oxidative stress, regulation of inflammatory responses (Magnusson et al., 1989; Gürer et al., 2001; Marcinkiewicz and Kontny, 2014; Altman et al., 2016), and in wound healing (Macalister, 1912; Eppler and Dawson, 2002; Araújo et al., 2010). Both the immune response and exposure to MA generate a strong oxidative environment within the human host. In this context, the first host response to this oxidizing environment is to increase antioxidant defense mechanisms. We speculate that increased antioxidant capacity in patients who respond to treatment, portrayed by higher levels of allantoin, taurin, and *N*-acetylglutamine, reflects higher oxidative stress, which in turn, could contribute to enhanced drug and ROS-mediated parasite killing, favoring therapeutic cure.

Allantoin, a downstream product of purine degradation, has been used as skin protectant in dermatological conditions (Government, U.S., 2010; Macalister, 1912; Madrazo-Jimenez et al., 2016). The oxidative stress induced upon exposure to MA could result in production of allantoin from purine degradation and urate interconversion. Higher abundance of taurine and allantoin in CL patients who cured, together with the evidence that allantoin promotes antimony-mediated parasite killing, concurs with their biological role as skin protectants and enhancers of wound healing, suggesting that in addition to serving as putative biomarkers of outcome, these metabolites could also contribute to drug-induced healing of CL through host-targeted mechanisms.

Leishmaniasis has been historically more frequently reported in adult males, primarily due to occupational exposure (Uribe-Restrepo et al., 2019). This contributes to one of the limitations of this study, which was the minimal inclusion of women for metabolomic profiling and the absence of children samples in the analyses. Considering the discovery nature of our study design, the target population was controlled as much as possible in terms of age, sex, ethnicity, and geographical region of origin to reduce demographic variability that could influence the metabolic profiles and that could skew candidate biomarkers selection. This limitation should be addressed in future validation studies using targeted metabolomics in larger patient cohorts that are age and sex matched, where the current data will serve as the basis for probabilistic estimation of the sample size.

Metabolic profiling of samples obtained by techniques of minimal invasiveness, such as collection of serum/plasma, urine, or saliva, facilitates the identification of biomarkers with a high potential to be translatable to the clinic. Implementation of metabolomics in neglected infectious disease research is just emerging, and most exploratory studies have been conducted in the context of African and American trypanosomiasis (Requena-Méndez et al., 2013; Vincent et al., 2016). Our discovery study reports the first plasma metabolomes of CL patients undergoing treatment with first-line MA, and provides the knowledge base for follow-up studies by establishing a set of candidate biomarkers to move forward to the validation, verification, and qualification phases of the pipeline of biomarkers of therapeutic responsiveness in CL.

Results presented here highlight the participation of metabolites mediating antioxidant and wound healing responses in clinical cure of CL, paving the way for the unexplored field of immunometabolomics, as a tool to inform novel therapeutics for leishmaniasis. Combination therapies of antileishmanial drugs and immunomodulators constitute one of the main approaches for novel antileishmanial therapies, seeking to reduce parasite burden while restoring immune homeostasis. A combination therapy of antimony and topical allantoin will constitute a next study phase, aiming to optimize antileishmanial treatment by reducing the exposure to toxic antimony doses, while promoting a healing response. Together, these results provide the research community with a set of well-defined candidate biomarkers for future validation, and constitute an important step toward urgently needed, clinically implementable methods to provide personalized medicine approaches for CL treatment.

## Ethics Statement

This study was carried out in accordance with the recommendations of Resolución número 8430 de 1993, Institutional Review Board for Ethical Conduct of Research Involving Human Subjects of CIDEIM with written informed consent from all subjects. All subjects gave written informed consent in accordance with the Declaration of Helsinki. The protocol was approved by the Institutional Review Board for Ethical Conduct of Research Involving Human Subjects of CIDEIM, approval code CIEIH 1209.

## Author Contributions

The authors researched, discussed, and approved the final version for submission. All authors made a significant intellectual contribution in experiment design, data analyses, and interpretation of data presented in the manuscript.

## Funding

This work was supported by US National Institutes of Health (NIH) grant R01AI104823 (https://www.niaid.nih.gov/) to MG, COLCIENCIAS grant 2229-65843177 contract no. 007-2015 to MG, and Newton-Caldas Fund grant 172715657 to RB and MG. DV and MP were supported, respectively, by COLCIENCIAS DSc student award 647 and Young Investigators and Innovators Program Contract Number 0552-2015.

## Conflict of Interest Statement

The authors declare that the research was conducted in the absence of any commercial or financial relationships that could be construed as a potential conflict of interest.
