# Supplementary material for: Pharmacometabolomics of Meglumine Antimoniate in Patients With Cutaneous Leishmaniasis
Source: Front Pharmacol. 2019 Jun 20;10:657. doi: 10.3389/fphar.2019.00657 (PMC6595045; doi:10.3389/fphar.2019.00657)
Supplement: Supplementary file 1 [file Presentation_1.pdf]

Supplementary Material

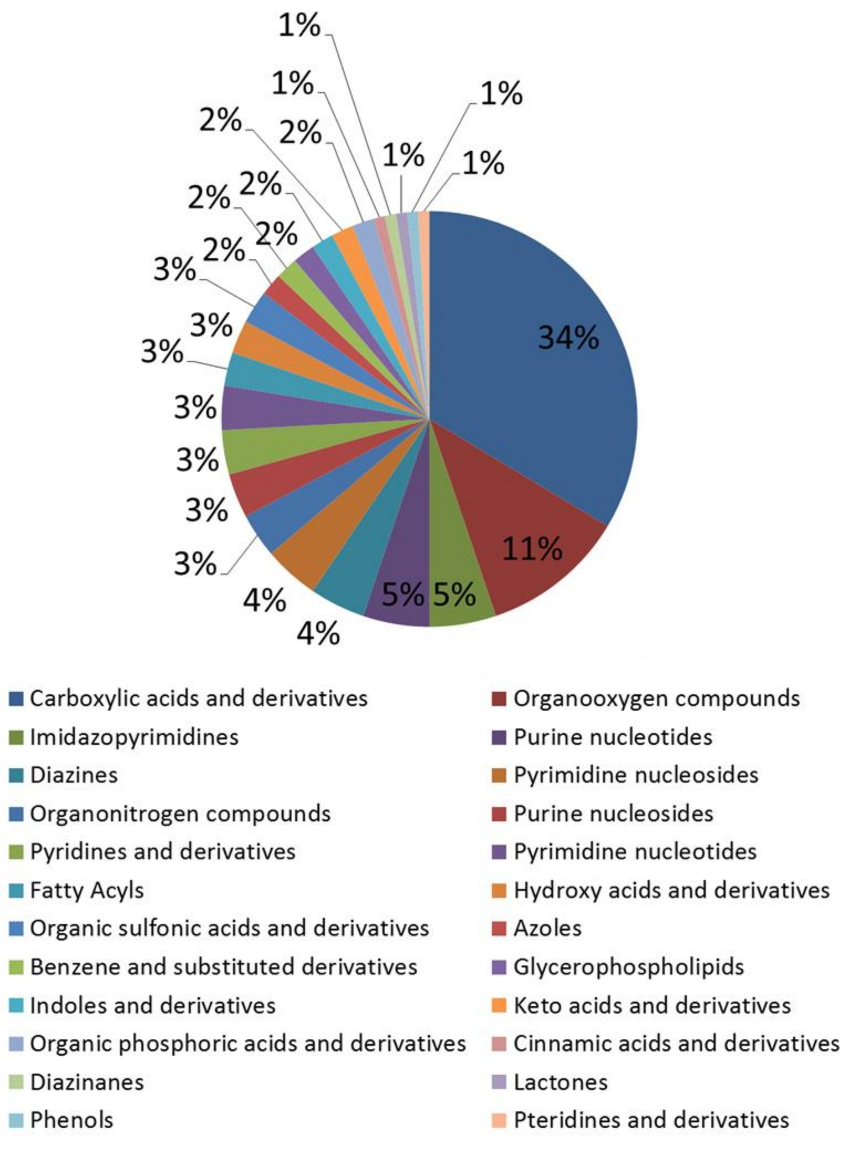

Supplementary figure S1. Reference metabolite standards. Pie chart shows the relative abundance of metabolite classes included as reference standards (n=116).

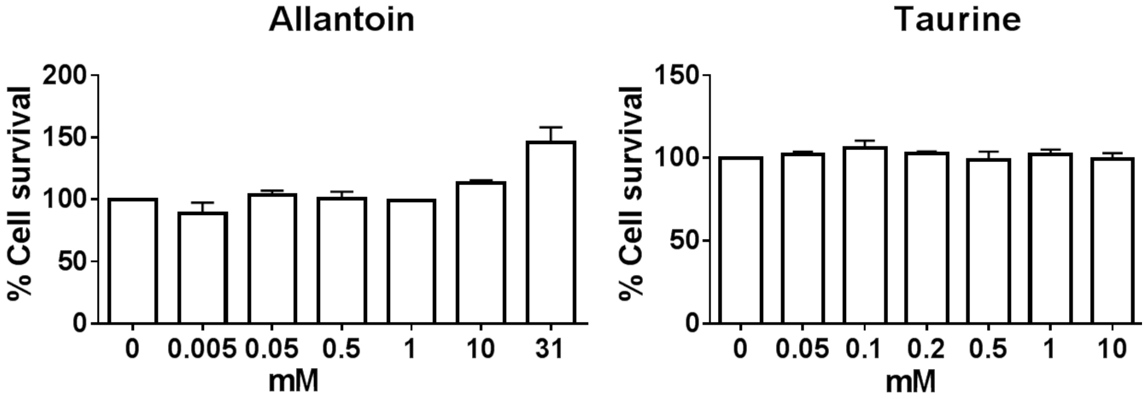

Supplementary figure S2. Allantoin and taurine cytotoxicity assay. The THP-1 human cell line was exposed to allantoin and turine and cell viability measured using the MTT assay. One hundred thousand cells were seeded in a 96 well plate in RPMI medium supplemented with 10% FBS and incubated at 37°C, 5% CO_2 with a dose range of allantoin or taurine for 72h. Data shown represent two independent experiments conducted in duplicate.

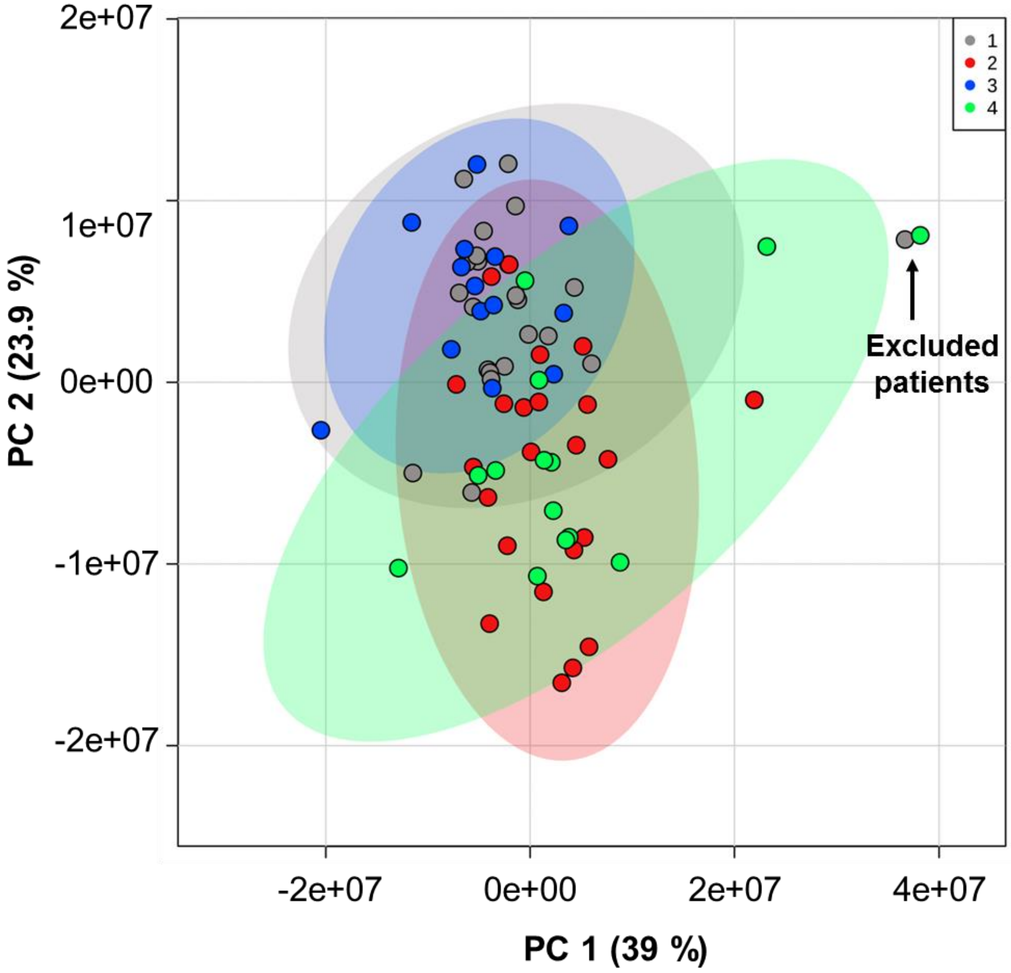

Supplementary figure S3. Principal component analysis plot of raw metabolite data. Each sample is represented by a single dot. Ovals represent the 95% confidence range. In grey, pre-treatment samples from responders; in red, post-treatment samples from responders; in blue, pre-treatment samples from non-responders; in green, post-treatment samples from non-responders.

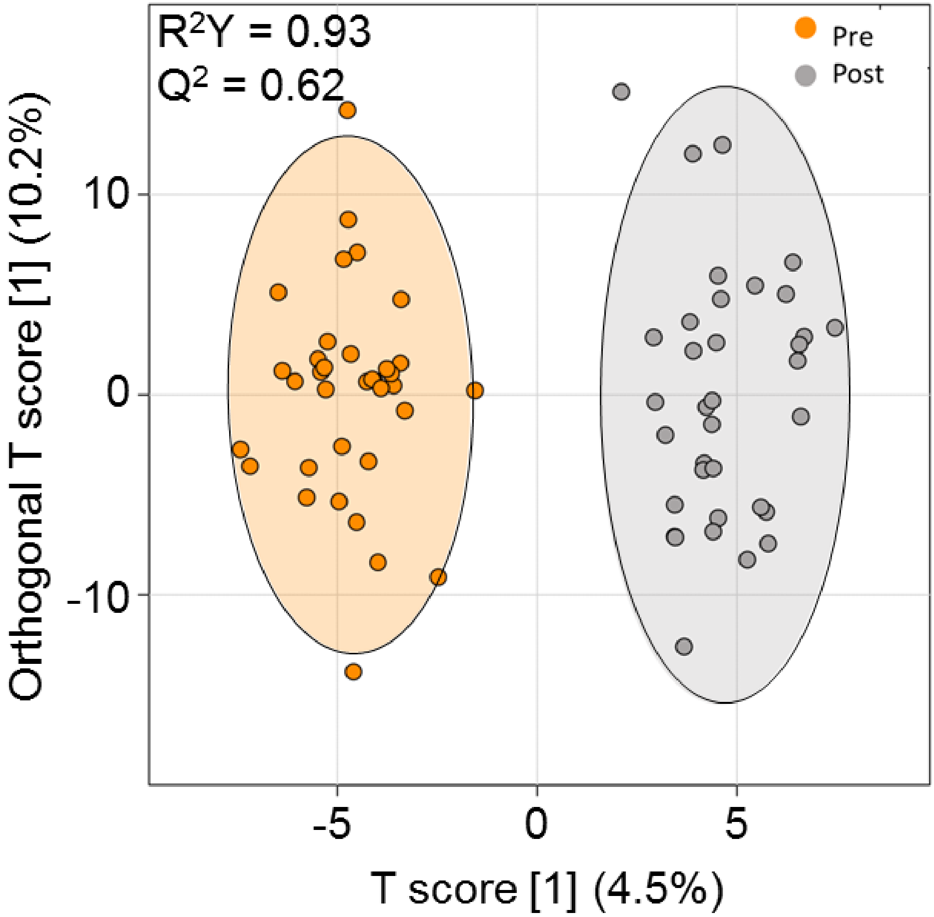

Supplementary figure S4. Scatter plot of the OPLS-DA model of plasma metabolomes of CL patients after MA exposure. Each sample is represented by an individual circle. In orange, samples obtained pre-treatment and in grey samples obtained at the end of treatment. T score [1] and Orthogonal T score [1] represent % variation that discriminates sample groups, and separation of samples within groups, respectively.

Dataset S1. Raw Metabolite counts. Patient codes indicate samples obtained pre or post treatment and the sequential number-identifier for each patient. Treatment outcomes are indicated in tab "ID Dataset S2". Mass in Daltons, RT- Retention time in minutes.

Dataset S2. Statistical analyses of pre vs post-treatment plasma samples. Contrast groups and type of statistical analysis are denoted in the top line of each column. Paired: paired statistical analyses; FDR: False discovery rate. Statistical significance was established as p value ≤ 0.05 and FDR ≤ 0.1.

Dataset S3. Statistical analyses of responders vs non-responders plasma samples. First tab show ANOVA Post-hoc Fisher´s LSD test. Ids: 1 - Responders pre-treatment, 2- Non-responders pre-treatment, 3- Responders post-treatment, 4- Non-responders post-treatment. Statistical significance was established as p value ≤ 0.05 and FDR ≤ 0.1. Second tab “OPLS-DA” show metabolites selected with a cut-off thresholds of $0.65 \leq p[1] \leq -0.65$ and $0.3 \leq p\ (corr)[1] \leq -0.3$ in the S-plot.
